# The Capabilities of Dedicated Small Satellite Infrared Missions for the Quantitative Characterization of Wildfires

**DOI:** 10.3390/jimaging8030078

**Published:** 2022-03-18

**Authors:** Winfried Halle, Christian Fischer, Dieter Oertel, Boris Zhukov

**Affiliations:** 1 German Aerospace Center (DLR), Institute of Optical Sensor Systems, Rutherfordstr. 2, 12489 Berlin, Germany; christian.fischer@dlr.de; 2 Astro- und Feinwerktechnik Adlershof GmbH, Albert-Einstein-Str. 12, 12489 Berlin, Germany; d.oertel@astrofein.com; 3 Space Research Institute, Russian Academy of Sciences, 84/32 Ul. Profsojusnaja, Moscow 117997, Russia; bzhukov@mail.ru

**Keywords:** fire detection, fire monitoring, fire attributes, infrared sensors, small satellites, fire radiative power (FRP)

## Abstract

The main objective of this paper was to demonstrate the capability of dedicated small satellite infrared sensors with cooled quantum detectors, such as those successfully utilized three times in Germany’s pioneering BIRD and FireBIRD small satellite infrared missions, in the quantitative characterization of high-temperature events such as wildfires. The Bi-spectral Infrared Detection (BIRD) mission was launched in October 2001. The space segment of FireBIRD consists of the small satellites Technologie Erprobungs-Träger (TET-1), launched in July 2012, and Bi-spectral InfraRed Optical System (BIROS), launched in June 2016. These missions also significantly improved the scientific understanding of space-borne fire monitoring with regard to climate change. The selected examples compare the evaluation of quantitative characteristics using data from BIRD or FireBIRD and from the operational polar orbiting IR sensor systems MODIS, SLSTR and VIIRS. Data from the geostationary satellite “Himawari-8” were compared with FireBIRD data, obtained simultaneously. The geostationary Meteosat Third Generation-Imager (MTG-I) is foreseen to be launched at the end of 2022. In its application to fire, the MTG-I’s Flexible Combined Imager (FCI) will provide related spectral bands at ground sampling distances (GSD) of 3.8 µm and 10.5 µm at the sub-satellite point (SSP) of 1 km or 2 km, depending on the used FCI imaging mode. BIRD wildfire data, obtained over Africa and Portugal, were used to simulate the fire detection and monitoring capability of MTG-I/FCI. A new quality of fire monitoring is predicted, if the 1 km resolution wildfire data from MTG-1/FCI are used together with the co-located fire data acquired by the polar orbiting Visible Infrared Imaging Radiometer Suite (VIIRS), and possibly prospective FireBIRD-type compact IR sensors flying on several small satellites in various low Earth orbits (LEOs).

## 1. Introduction

Fire activity is a global phenomenon characterized by strong spatial and temporal variability. Wildfires are an important disturbance to the ecosystem, resulting in land cover alteration and change, and atmospheric emissions on multiple time scales. Catastrophic wildfires in recent years have again given a clear message that, in the deployment of limited suppression resources, sound fire management decisions (including justifications of a possible severity ranking) rely very much on timely and detailed information on the location, intensity, direction and rate of spread of the fire fronts.

In general, Earth observation information on wildfire activity, among others, is used for:

- Global climate change research, estimating atmospheric emissions and developing periodic global and regional assessments (i.e., quantification of the fire impact on climate);
- Fire management and ecosystem management planning, including post-fire recovery efforts and wildland management;
- Operational purposes (preparedness and wildfire suppression);
- The development of informed policies.

Satellite Earth observation is appropriate to provide repetitive data at spatial and temporal scales necessary for the detection and monitoring of wildfires [1].

“Fire Disturbance” is one of several land-oriented Essential Climate Variables (ECVs) defined by the Global Terrestrial Observing System (GTOS), and this ECV comprises three sub-variables: Burned Area, Active Fire Detection (i.e., locating active fires) and Fire Radiative Power (FRP).

This “trinity” reflects, among other things, data availability and the “growing process” of deriving higher-quality fire data products for estimating carbon emissions from vegetation fires:

- There have been and continue to be many satellite sensors whose imagery data are suitable for determining burned areas from wildfires. However, fire emissions can only be estimated from the burned area data with uncertainty.
- There are a major number of satellite sensors whose image data in the MIR band allow for the detection of active fires. However, their MIR band data are often not suitable for quantitative characterization of the fires, in part due to saturation effects in their signal tracts.
- There are only a few infrared satellite sensors that:
  (i) Do not suffer from saturation effects when recording extended and flaming 1000 K (727 °C) hot fires;
  (ii) Simultaneously resolve the temperature of the “cold” backgrounds (of the fires) with an accuracy of or better than 0.5 K.

Only data from the MIR and TIR bands of these few infrared satellite sensors allow the derivation of quantitative fire attributes, such as the FRP.

From the FRP, carbon emissions from the fires can be estimated with much higher accuracy than from, for example, the burned area datasets [1].

Comment: Multiple FRP observations of a fire cluster and the consecutive time integration of these FRP values can provide an estimation of the Fire Radiative Energy (FRE), which is a direct measure of the total CO_2_ emitted by the fire during the whole observation period.

The ratio of the FRP of a single fire front to its length is called the Fire Line Strength (FLS), which is a measure of the fire’s severity. The FLS is directly related to the fire’s impact on the ecosystem.

There are two crucial requirements for IR sensor (the term “IR sensors”, as used in this paper, stands for imaging infrared sensors with at least one spectral band in the mid-IR band (MIR at 3.8 µm) and one spectral band in the thermal IR band (TIR, between 8 and 12 µm)) data to be used for the derivation of the FRP:

- The signal must be free from saturation effects;
- The radiometric precision of the sensor must allow for the distinguishment between pixels occupied by fires, even small ones, and “background pixels not touched” by fires.

No civil space-borne IR sensor fulfilled this requirement before the year 2000 because the Moderate Resolution Imaging Spectroradiometer (MODIS) on the “Terra” satellite, the first system which fulfilled this requirement, was launched at the end of 1999.

The small satellite Bi-spectral Infrared Detection (BIRD) was developed and built by the German Aerospace Center (Deutsches Zentrum für Luft unf Raumfahrt (DLR)) and successfully operated from October 2001 until February 2004 [2,3].

The small German satellites Technologie-Erprobungs-Träger N° 1 (TET-1) and Bi-spectral InfraRed Optical System (BIROS) were piggyback launched in July 2012 and June 2016, respectively. Both satellites are part of the space segment of the FireBIRD mission, led by the DLR [4,5].

The IR sensors of BIRD, TET-1 and BIROS provided image data in the mid-IR (MIR, at 3.8 µm) and thermal IR (TIR, at 9.2 µm) bands, with ground sampling distances (GSD) of ~180 m in both spectral bands, and noise equivalent difference temperatures (NEDTs) of 0.4 K (at 300 K) in the MIR band and 0.2 K (at 300 K) in the TIR band. Both bands are also composed of a very high radiometric dynamic, necessary to avoid signal saturation from strong high-temperature events (HTEs), such as big wildfires or major volcanic eruptions. The combination of these features allows for the estimation of fire attributes such as the fire temperature, the fire area or the FRP of small and huge vegetation fires, as explained in detail in Chapters 4 and 5 of [3]. These unique features of the push broom-type MIR/TIR sensor of BIRD, TET-1 and BIROS, called the “Hot Spot Recognition System (HSRS)”, are achieved by the real-time adjustment of their integration time: if, after sampling with the regular exposure time, the real-time sensor onboard data handling indicates that the signals of some detector elements are saturated (or close to saturation), a second exposure is performed within the same sampling interval, but with an exposure time to be reduced by an order of magnitude, i.e., by a factor of ~12. The data of both exposures are digitized and stored by the onboard data handling system. After data transmission to the ground station, the data samples with these double exposures are merged during the on-ground processing. This smart “Hot Area (HA) reconstruction” procedure preserves the 0.4 K and 0.2 K radiometric resolution in the MIR and TIR channels, respectively, and eliminates detector saturation for huge wildfires and strong volcanic lava eruptions [5,6].

These capabilities of BIRD and FireBIRD are a unique feature compared with other existing IR sensors, i.e., imaging infrared sensors with at least one spectral band in the mid-IR band (MIR at 3.8 µm) and one spectral band in the thermal IR band (TIR, between 8 and 12 µm), currently used for HTE observations.

However, imaging IR sensors with one or more spectral bands in TIR, but without a band in MIR, are not capable of providing data for the estimation of the above-mentioned fire attributes. The MIR band is used as the “leading channel” for the detection of active fires and their quantitative analysis. This is mainly due to the fact that the signal contrast of fire-occupied pixels to “cold” background pixels is much higher in the MIR band than in the TIR band, which is illustrated in Figure 4 of [3]. Furthermore, an MIR band NEDT of <0.5 K (at a 300 K blackbody temperature) is a very important prerequisite for its “leading channel” function.

One objective of this paper was to demonstrate the capability of BIRD- and FireBIRD-type IR sensors with higher spatial resolutions (<300 m) and radiometrically accurate MIR and TIR bands (with NEDTs < 0.4 K) that do not suffer from saturation effects caused, for example, by large fires. This is conducted in Section 2 of this paper, showing three examples of quantitative characterizations of active fires, comparing BIRD and FireBIRD data with the data of IR sensors on operational polar orbiting satellites acquired nearly simultaneously.

Further objectives of the paper were as follows:

(i) To illustrate the growing role of new geostationary IR sensors in fire observation;
(ii) To simulate the fire recognition potential of the upcoming Meteosat Third Generation-Imager (MTG-I);
(iii) To predict the degree of synergy, i.e., the possible improved quality of the observation and quantitative evaluation of fires, especially in their diurnal cycle, if data from respective geostationary IR sensors, polar orbiting IR sensors and FireBIRD-type IR sensors are combined.

These three issues, i.e., items (i)–(iii), are the main content of Section 3, Section 4 and Section 5 of this paper, respectively.

## 2. Selected Application Examples—Using Data of BIRD, FireBIRD and Operational Polar Orbiting Satellite IR Sensors

### 2.1. Main Features and Parameters of Operational Polar Orbiting IR Sensors

This chapter shows selected application examples where data from BIRD, TET-1 and BIROS are used in comparison with IR sensor data registered nearly simultaneously by operational satellite systems. Table 1 shows features and parameters of three operational IR sensors: Moderate Resolution Imaging Spectroradiometer (MODIS), Visible Infrared Imaging Suite (VIIRS) and Sea Land Surface Temperature Radiometer (SLSTR), in comparison with the Hot Spot Recognition System (HSRS), flown on BIRD, TET-1 and BIROS.

The BIRD demonstrator mission as well as the FireBIRD mission with the two satellites TET-1 and BIROS was defined, developed and operated as a science mission. BIRD and FireBIRD were operated on user demand and did not generate data with a duty cycle of 100%, i.e., in “24/7” operation, as it is the case for the operational IR sensors MODIS, VIIRS and SLSTR.

An important parameter of sun-synchronous orbiting satellites is the local time of their upward or downward equator crossing, the so-called *Local Time of Ascending Node* (LTAN) or *Local Time of Descending Node* (LTDN), respectively.

The LTAN and/or LTDN can be used to find out the observation time difference of various satellites. Table 2 shows the LTAN and LTDN of the operational satellites carrying the IR sensors MODIS, VIIRS and SLSTR, as well as the LTAN and LTDN of BIRD, TET-1 and BIROS. This overview permits a “grouping” N°, considering possible years of common utilization and low observation time differences.

The selection of the LTAN and LTDN of the operational satellites carrying MODIS, VIIRS and SLSTR was driven by the requirements of a great number of applications. Their orbits are not optimized for fire detection. In particular, the NOAA-20 (VIIRS) system with its LTAN at 13:30 h must fulfill the requirements of a great number of important and higher-ranking utilizations than fire applications, such as ocean color analysis [7]. The LTAN and LTDN of the operational satellites, mentioned in Table 2, are kept stable by orbit maintaining using propulsion systems.

Due to the better availability of affordable and well-timed piggyback launch opportunities (worldwide) into LEO for the dedicated fire detection satellites BIRD, TET-1 and BIROS, morning orbits resulted for all satellites. The morning orbits, however, allow the detection of so-called incipient fires early in the day, i.e., the anti-meridiem detection of small HTEs, representing a further “unique selling point” of BIRD and FireBRID. However, in this sub-chapter and in Table 2, we do not aim at “orbit optimization for fire detection”, but we look for orbit communalities only with respect to possible years of common utilization and low observation time differences.

The LTAN and LTDN of the piggyback launched BIRD, TET-1 and BIROS initially were all close to the LTAN and LTDN of the main satellites of these launches. The LTDN of BIRD and the LTAN of TET-1 drifted as indicated in Table 2. They could not be corrected because they had no propulsion system to correct their local times of equator crossing.

In the right column of Table 2, “grouping” numbers for comparing the utilization of IR data registered nearly simultaneously by operational satellite systems, on one hand, and data of BIRD or FireBIRD, on the other, are presented.

Table 3 shows an overview of selected scientific papers and scientific documents, called “References”, structured by the “grouping” numbers presented in the right column of Table 2.

Comment: Many scientific papers describe the BIRD, TET-1 or BIROS system function and the utilization of BIRD or FireBIRD data without comparison to other data sources, but this chapter contains selected application examples based on data registered nearly simultaneously by operational satellite systems and by BIRD or FireBIRD.

The following examples for the combined utilization of data registered nearly simultaneously by operational satellite systems and by BIRD or FireBIRD were selected:

- MODIS “Terr”–BIRD data comparison in a fire scene in Siberia close to Lake Baikal—see [9], according to “grouping” N° 1 (in Table 3);
- MODIS “Aqua”–TET-1 data comparison in low-temperature peat fire scenes in Borneo—see (Atwood et al., 2016), according to “grouping N° 2”;
- TET-1–BIROS-VIIRS “Suomi NPP” data comparison for the devastating “Paradise fire” in California/the US—see [5], according to “grouping N°3”.

### 2.2. Two Methods to Derive the Fire Radiative Power (FRP)

The Fire Radiative Power (FRP), a very useful parameter for the characterization of the amount of burnt vegetation and of gas and aerosol emissions released by a fire, can be assessed, as introduced in Section 1 of this paper, by two methods, which are explained in detail, for instance, in [3]:

- A method for FRP estimates, using the effective fire temperature T_F_ and effective fire area A_F_, both derived from MIR and TIR channel data (this FRP estimate can be used for flaming fires and for smoldering fires with T_F_ < 700 K):
  FRP = σ (T^4^_F_ − T^4^_bg_) A_F_ (1)
  where σ is the Stefan–Boltzmann constant, and T_bg_ is the background temperature;
- The “Wooster method” for FRP* estimates, using the MIR channel signal only (with sufficient accuracy only for flaming fires with temperatures > 700 K):
  FRP* = 4.35 P_F_ (L_mir_ − L_mir-bg_) (2)
  where P_F_ is the MIR ground pixel area, and L_mir_ and L_mir-bg_ are the BIRD MIR radiances of a hot pixel and of the background, respectively.

### 2.3. MODIS–BIRD Data Comparison in a Fire Scene in Siberia Close to Lake Baikal

For a fire scene in Siberia close to Lake Baikal, the quantitative fire parameters were retrieved from the BIRD and MODIS data in [8] using the same methodology in order to provide directly comparable results. The methodology included the following steps:

- Consolidation of contiguous hot pixels in hot clusters;
- Retrieval of the effective fire temperature, effective fire area and FRP for the hot clusters;
- Estimation of the TIR background temperature variability (background clutter) and of the confidence intervals for the effective fire temperature, effective fire area, FRP and pronounced fire fronts;
- Estimation of the front length and radiative intensity (ratio of the FRP to the front length, characterizing the fire front strength).

**Figure 1:**
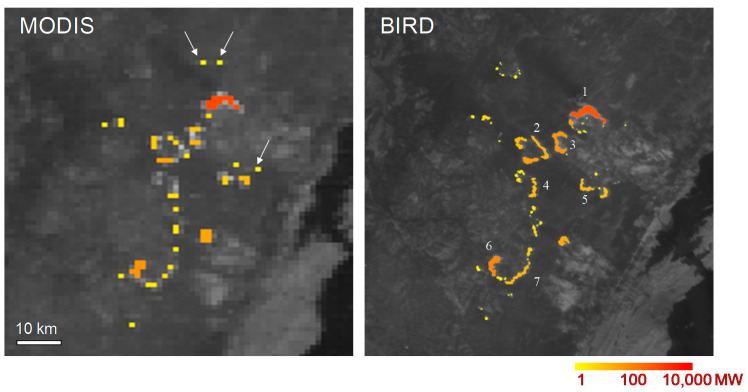
Fragments of forest fire images at Baikal, obtained by MODIS and BIRD on 16 July 2003, with a time interval of 25 min. Images show the MIR image from each sensor, and detected hot clusters are color coded according to their Fire Radiative Power (FRP) in megawatts (MW). The arrows in the MODIS image fragment show probable false alarms (because real fires could hardly cool down sufficiently in the short time interval between MODIS and BIRD that the data take not to be seen as hot spots in the higher-resolution BIRD fire scene fragment).

Figure 1 shows an area west of Lake Baikal, Russia, observed nearly simultaneously, i.e., within a 30-minute interval, by MODIS/“Terra” and BIRD on 16 July 2003. The comparison clearly shows that only the BIRD data allow an estimation of fire front attributes relevant for fire managers. MODIS MIR and TIR data have a 1 km ground resolution at the nadir. They do not allow estimating fire front attributes, except for the FRP. This comparison and the figures obtained from BIRD data only, presented in Table 4, provide a useful metric by which the enhanced performance of BIRD in terms of fire detection and characterization can be judged [9].

MODIS data allow the detection of larger fire fronts or only separate hot spots where one cannot recognize clear fire fronts. In contrast, the higher-spatial resolution BIRD data make it possible to recognize larger fire fronts and to estimate their attributes: FRP, fire front length, fire front strength and fire front effective depth.

### 2.4. MODIS “Aqua”–TET-1 Data Comparison in Low-Temperature Peat Fire Scenes in Borneo

Peatlands naturally have a high water table, lying at or just below the forest-covered surface. Drainage infrastructure, such as canals, can contribute to lowering the water table, which is then compounded by drought periods coinciding with climatological events such as El Niño–Southern Oscillation (ENSO). The reduced water table allows drying of the peat layer, often for the first time in centuries, thus becoming more susceptible to catching fire. Fire is often utilized as a cheap, effective method to clear and maintain land for both agricultural and plantation development. In Borneo, slash-and-burn techniques often result in fires spreading into surrounding un-slashed peat swamp forests. Peatland fires are characterized by low-intensity burning, which can spread into peat deposits up to 0.5 m below the surface and can burn for long periods of time, often being very difficult to extinguish. Smoldering peatland fires produce large amounts of particulate matter, CO and other gas compounds [10].

**Figure 2:**
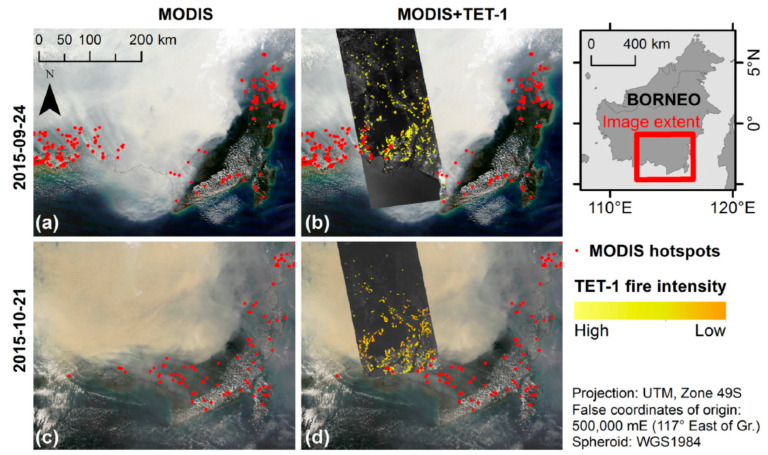
Mosaic of MODIS “Aqua” and TET-1 image fragments obtained on 24 September 2015 (**a**,**b**) and on 21 October 2015 (**c**,**d**) over Borneo’s region indicated in top right. Red dots are detected hot spots based on MODIS data. The intensity scale of numerous peat fires derived from TET-1 data is also shown on the right [10].

Figure 2 shows MODIS images and hot spot data compared with a TET-1 imagery overlay from [10]:

N° (a)—a MODIS “Aqua” true color image from 24 September 2015, with the smoke from the fires (the smoke is dominating in the true color images, i.e., in the 0.4–0.65 wavelength region; the smoke is nearly transparent in the 3.6–4 µm wavelength region, as can be seen in the center MIR band stripe of Figure 2b,d), superimposed with same-day MODIS hot spot data (red dots).
N° (b)—The MODIS image N° (a) overlaid with a same-day TET-1 MIR band image fragment.
MODIS hot spot data appear to omit low-intensity fire fronts visible in the TET-1 imagery (fire intensity is indicated by the yellow gradient).
N° (c)—a MODIS “Aqua” true color image from 21 October 2015, superimposed with same-day MODIS hot spot data.
N° (d)—the MODIS image N° (c) overlaid with a TET-1 MIR band image fragment, which shows MODIS hot spot active fire detection being inhibited by thick smoke and haze.

**Figure 3:**
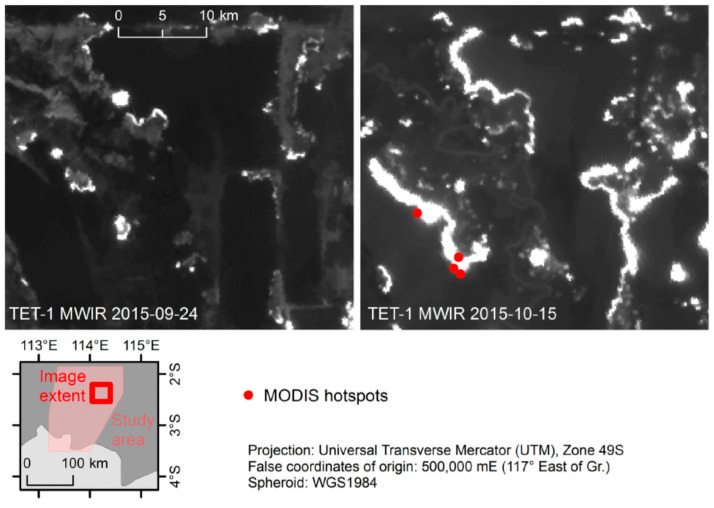
TET-1 MIR band image fragments from 24 September and 15 October 2015 showing the rapid fire front growth over 2 weeks. Red dots show the small number of active fires registered by MODIS nearly simultaneously with TET-1 [10].

Figure 3 illustrates the rapid peat fire front growth over two weeks showing two TET-1 MIR band image fragments from 24 September and from 15 October 2015. Red dots show—in contrast—the small number of active peat fires detected nearly simultaneously by MODIS “Aqua”. The location of the image extent within the study area is indicated in the lower inset of Figure 3.

This example demonstrates the improved FireBIRD fire detection and analysis capabilities compared to MODIS due to its IR sensor’s higher saturation temperature and finer spatial resolution (see Table 1).

### 2.5. BIROS–VIIRS “Suomi NPP” Data Comparison for the Devastating “Paradise Fire” in California/US

The devastating “Paradise” bushfires in California, USA, were observed by FireBIRD between 10 and 14 November 2018.

**Figure 4:**
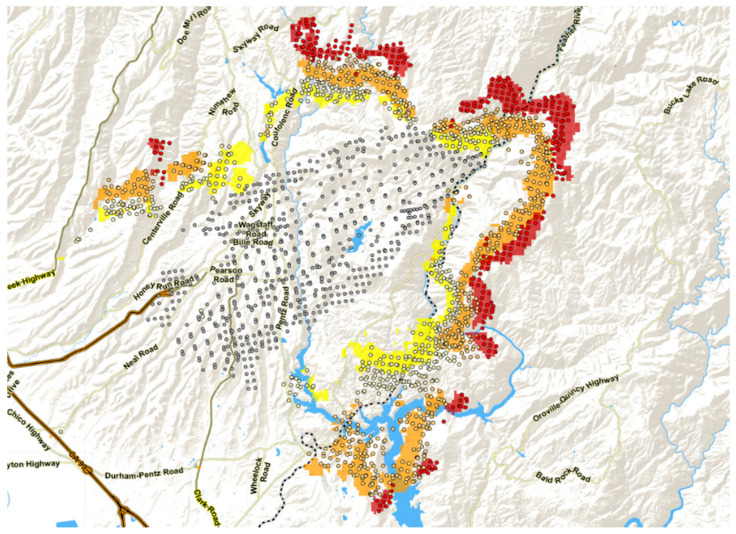
Yellow-, orange- and red-colored “Paradise” fire fronts, observed by VIIRS and TET-1 on 10, 12 and 14 November 2018, respectively. The non-colored small circles in the center stem from VIIRS data obtained on 8 November 2018.

**Figure 5:**
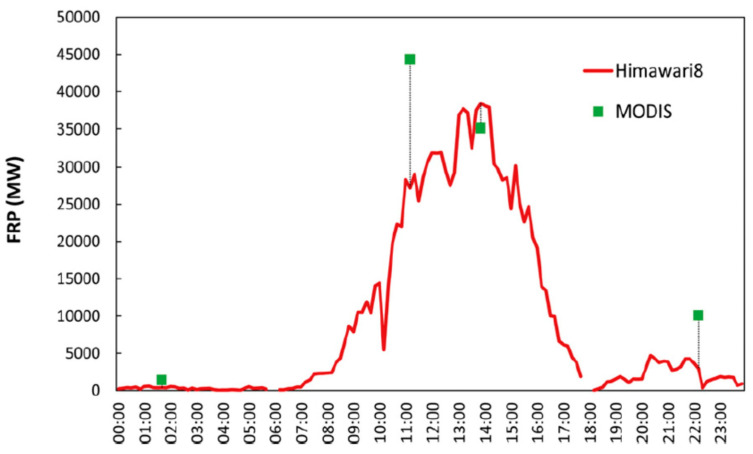
FRP diurnal cycle of agricultural residue burning in eastern China on 11 June 2015 derived from Himawari-8 and MODIS data [13].

Figure 4 shows the movement of the “Paradise” fire fronts in a map projection of three FireBIRD data-takes. This map projection was developed by the DLR “Zentrum für satellitengestützte Kriseninformation”, i.e., the Center for Satellite Based Crisis Information, to support the decisions of the regional authorities and the fire fighters. The small circles in Figure 5 show the pixel centers of VIIRS band I-4 records conducted from 8 to 14 November 2018. An excellent coincidence of the fire front movement observed by FireBIRD and VIIRS can be found in Figure 4 (the non-colored small circles in the center of Figure 4 stem from VIIRS data acquired on 8 November 2018, when FireBIRD could not observe the “Paradise” fire).

Table 5 shows the following fire attributes of the “Paradise” fire for five days in November 2018, derived from FireBIRD data acquired for the fire zone shown in Figure 4 for the indicated dates and local times:

- The aggregated FRP, i.e., the summarized Fire Radiative Power of all fires given in megawatts (MW);
- The mean effective fire temperature T_F_, i.e., the calculated mean value of the effective fire temperature of all fires, in kelvin (K);
- The aggregated effective fire area A_F_, i.e., the summarized burning area of all fires, in hectares (Ha);
- The number of MIR band pixels occupied or “infected” by fire of all fires (“occupied” means that the fire pixel area is, as the rule, partly filled with fire; N° 1 is the difference from the background pixels, which are not touched by fire);
- The aggregated area of all MIR band pixels occupied by fire P_F_, i.e., the summarized pixel area of all pixels “infected” by fire, in Ha;
- The ratio k of the aggregated effective fire area A_F_ to the aggregated area of all MIR band pixels occupied by fire P_F_.

The values of the fire attributes are presented in Table 5 for the whole fire scene, which is, by the way, quite different to the content of Table 4, where the fire attributes are shown for the single fire clusters. However, the estimates of the fire attributes in both tables were derived from the sub-pixel domain.

**Table 5:** Fire attributes of the “Paradise” fire for five days in November 2018, derived from four TET-1 records and two BIROS records.

Date	10.11.	10.11.	12.11.	14.11.	21.11.	22.11.
Local time	00:19 h	13:09 h	13:14 h	13:19 h	21:18 h	10:03 h
Acquired by	TET-1	TET-1	TET-1	TET-1	BIROS	BIROS
Aggregated Fire Radiative Power/FRP/ (MW)	2792	3327	4953	1089	69.7	3.1
Mean effective fire temperature—T_F_ (K)	714	672	658	752	720	891
Aggregated effective fire area—A_F_ (Ha)	20.2	31.5	52.0	88.3	0.0191	0.0052
Number of the occupied by fire MIR-pixels	1511	1738	2241	1173	188	17
Aggregated area of all by fire occupied MIR-pixels— P_F_ (Ha)	4366.79	5022.82	6476.49	3389.97	543.32	49.13
Mean area ratio k = A_F_/P_F_	0.0046	0.0063	0.0080	0.026	0.0018	0.011

The colors of the three columns in Table 5 coincide with the colors of the fire fronts in Figure 4.

It also seems interesting to mention that in the “Paradise” fire scene, the aggregated effective fire area A_F_ is mostly two orders of magnitude lower than the aggregated area of all MIR pixels occupied by fire P_F_.

The area ratio of k = A_F_/P_F_ quantifies the mean value of the area of MIR pixels partly occupied by the detected fires in the observed scene. As the numbers in the lower line of Table 5 show, the values of k vary between 0.0018 (0.18%) and 0.026 (2.6%).

According to these considerations, it is relevant to define the maximum possible k_max_ indicating the upper threshold of MIR pixel occupation by fire; beyond this—due to the Stefan–Boltzmann law (the Stefan–Boltzmann law states that the total radiation of a blackbody is proportional to T^4^, where T is the temperature of the blackbody, given in K)—signal saturation may occur. Considering the high signal contribution of the hot area parts in fire-occupied pixels, a rough approximation (using two temperatures: T_pix max_, the maximum blackbody temperature in K when MIR pixel saturation may occur, and T_fire max_, the maximum fire temperature expected in the scene in K) can be proposed for k_max_:

k_max_ = [T_pix max_/T_fire max_]^4^ = A_Fmax_/P_F_ (3)

This is true, because the signal of a pixel, generated by the product of P_F_ × T^4^_pix max_, is equal to the signal of the product of A_Fmax_ × T^4^_fire max_.

Table 6 presents the following physical values and figures for the MODIS MIR band 22, the HSRS (on BIRD, TET-1 and BIROS) MIR band, the SLSTR MIR band F1 and the VIIRS (on Suomi NPP and NOAA-20) MIR band M-13:

- Values of the nadir ground pixel sizes;
- The designed T_pix max_, i.e., the maximum temperature of an observed blackbody which would not lead to pixel signal saturation;
- The calculated k_max_, i.e., the value of the maximum area in the MIR pixels which are partly occupied by a flaming fire with a temperature of 1000 K in the observed scene;
- The derived maximum figures for (assuming a maximum fire temperature, expected in the fire scene of T_fire max_ = 1000 K):
  (i) The area of a 1000 K fire in a ground pixel;
  (ii) The fire front depth, crossing the nadir ground pixels. The mean area ratio k = A_F_/P_F_ = 0.026 in the red-colored column of Table 5, calculated for 360 m × 360 m FireBIRD MIR band pixels, is greater than k_max_ = 0.0208 for an assumed T_fire max_ = 1000 K in the two “VIIRS”-related cases of Table 6. In other words, this means, if the FRP were derived for the “Paradise” fire scene from 14 November 2018 using the VIIRS M-13 band data with a 375 m × 375 m pixel size, errors might occur due to pixel saturation.

**Table 6:** Selected parameters and figures of fire application MIR bands of the operational sensors and the HSRS, presented in Table 1 for: (a) the nadir ground pixel sizes, (b) the T _pix max_ specified for the instrument, (c) the k_max_ calculated by Relation (1), and (d) the derived maximum figures for (d, i) the area of an assumed 1000 K fire in a ground pixel and (d, ii) the depth of an assumed fire front crossing the nadir ground pixels. The following column colors were selected for the saturation probabilities: green for low, gray for medium and pink for high.

Parameter/Figure	MODIS	VIIRS		SLSTR	HSRS
(a) Ground pixel size at the nadir (m^2^)	1 km × 1 km = 1,000,000	375 m × 375 m = 140,625	750 m × 750 m = 562,500	1 km × 1 km = 1,000,000	360 m × 360 m = 129,600
(b) T_pix max_, (K)	450	380	380	500	630
(c) k_max_ for an assumed T_fire max_ = 1000 K	0.0410 (4.1%)	0.0208 (2.08%)	0.0208 (2.08%)	0.0625 (6.25%)	0.1575 (15.75%)
(d, i) Maximum area A_Fmax_ of an assumed 1000 K fire in the ground pixel (without saturation)	41,000 m^2^	~3000 m^2^	12,000 m^2^	62,500 m^2^	20,500 m^2^
(d, ii) Maximum depth of a 1000 K fire front crossing the ground pixel	~30 m	~6 m	~24 m	~44 m	~40 m

If the regular VIIRS M-band active fire product data (see https://viirsland.gsfc.nasa.gov (accessed on 1 December 2021)), with a 750 m × 750 m pixel size in the swath +/− 31.5° around the nadir, were used for the estimation of the FRP in the “Paradise” fire scene from 14 November 2018, the derived FRP values would probably not be disturbed by saturation errors.

New generations of geostationary meteorological satellites now make it possible to record the diurnal cycle of fires, beginning from medium fires with FRP figures of ~50–100 MW upward [13]. The combination of these frequently provided coarser-spatial resolution data with data from the above-described operational polar orbiting IR sensors and higher-resolution FireBIRD-type data will offer a new quality in climate change-related carbon emission investigations.

## 3. Example of FireBIRD and Himawari-8 Fire Scene Observation

The Japanese geostationary meteorological satellite Himawari-8, launched in 2015, was the first satellite worldwide which provided mid and thermal IR data with a 2 km ground sampling distance (GSD) at its sub-satellite point (SSP), and a repetition period of 10 min also usable for fire-related applications, including the estimation of the FRP.

Geostationary active fire detection has previously been provided over Asia and Australia using Japanese Multifunctional Transport Satellite (MTSAT) imagery and the Korean Communication, Ocean and Meteorological Satellite (COMS), though FRP assessment has typically not been available [13].

The Advanced Himawari Imager (AHI) spectral band 7 at 3.8 µm and band 14 at 11.1 µm both have a GSD of 2 km at their SSP, which is significant progress compared, for instance, to the MTSAT, with a GSD of 4 km at its SSP in its 3.8 µm and 11 µm bands.

The imager of the US Geostationary Orbiting Environmental Satellite (GOES-R), launched in 2017, also has a 2 km GSD at its SSP in these bands.

MIR and TIR data from Himawari-8 and GOES-R significantly improve the observation accuracy with regard to the dynamic nature of fires in their diurnal cycle, which is illustrated in Figure 5, showing the FRP diurnal cycle of agricultural residue burning in eastern China on 11 June 2015 derived from Himawari-8 and MODIS data [13].

**Figure 6:**
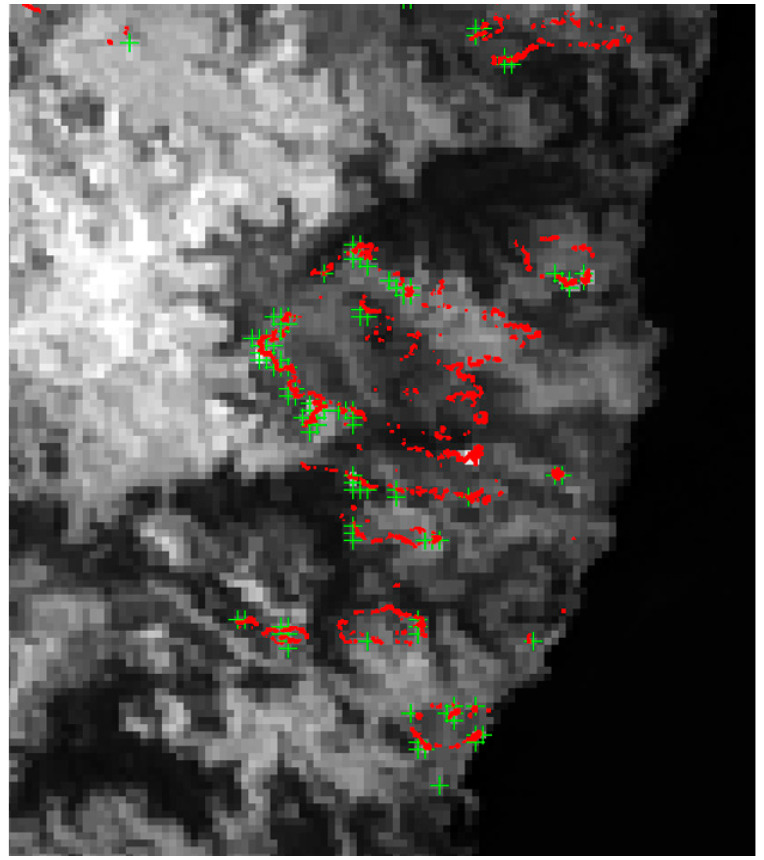
An image fragment of the AHI-band 7, acquired near Sydney on 10 November 2019 at 10:10 h local time. The green crosses mark fires detected in this AHI band. The red fire clusters were derived from BIROS IR data recorded over the same region only minutes later.

It is also of interest to compare fire data obtained by Himawari-8 and FireBIRD. Figure 6 shows an Advanced Himawari Imager (AHI)-band 7 image fragment of a fire scene in eastern Australia near Sydney, recorded on 10 November 2019 at 10:10 h local time with green crosses for the fires detected in this band and with an overlay of red fire clusters derived from BIROS IR data recorded over the same region only minutes later.

The GSD of the AHI-band 7 (at 3.85 µm) in this region is 2.1 km × 2.5 km (east–west × south–north), which also represents the pixel size in the image fragment. Most of the active fires detected in this AHI-band 7 image fragment are located on or close to the major fire clusters derived from the BIROS IR data.

Figure 6 also illustrates that smaller or weaker fire clusters derived from the BIROS data are omitted in the AHI active fire list. This is due to the 2.1 km × 2.5 km ground pixel size of the AHI-band 7, which allows detecting only flaming fires with an effective fire area greater than 500 m^2^, whereas the BIROS pixel size of 0.36 km × 0.36 km permits the detection of flaming fires with a minimum effective fire area of 15 m^2^. This is due to the fact that the area proportion of a flaming fire in an MIR pixel must be >1.0 × 10^−4^ to be reliably detected, which is explained in detail, for instance, in [3].

## 4. Simulation of the Meteosat Third Generation Imager Fire Channel Potential

The third generation of European geostationary meteorological satellites is currently being prepared by the ESA, EUMETSAT and their industrial partners, aiming at a launch date in 2022.

**Figure 7:**
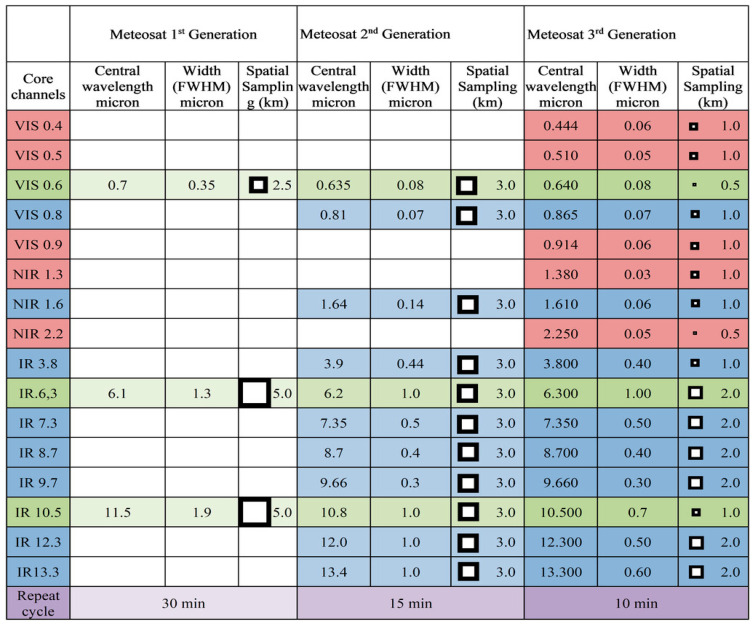
Summary of the growth of imaging capabilities from the Meteosat First Generation (MFG) through the Second to the Third Generation [14].

The Flexible Combined Imager (FCI) of the Meteosat Third Generation Imager (MTG-I)—also capable of fire-related applications—will provide mid and thermal IR data with a 1 km or 2 km GSD at its SSP with a repetition period of 10 min. The FCI instrument will continue the successful mission of the SEVIRI instrument on the Meteosat Second Generation (MSG). The FCI has additional channels and improved spatial, temporal and radiometric performance, compared to the current SEVIRI. The requirements for the FCI are driven by regional and global numerical weather prediction (NWP) and nowcasting. The FCI design allows a Full-Disk Scan Service (FDSS), with a basic repeat cycle of 10 min, and an alternative fixed European regional Rapid-Scan Service (RSS), which covers one-quarter of the full disk with a repeat cycle of 2.5 min. It is planned to provide this dual capability once two MTG-I satellites have been successfully deployed, with one satellite dedicated to FDSS and one to RSS. Figure 7 provides a summary of the growth of imaging capabilities from the Meteosat First Generation (MFG) through the Second to the Third Generation. It shows how the spectral capabilities have increased while retaining the capabilities of the previous generation. The inserted squares depict the improved spatial resolution at the sub-satellite point in a comparative manner. The corresponding temporal repeat cycles for full-disk imaging for the three Meteosat generations are 30, 15 and 10 min, respectively [14].

MTG-1 is foreseen to be launched at the end of 2022. Table 7 shows the characteristics of the MTG-I/FCI fire application channels, reprinted with permission from [14].

In this paper, it is reasonable to present the simulation results of the MTG-I/FCI fire application MIR and TIR channels, including their expected fire detection and analysis capability, using image sets obtained by the Bi-spectral IR Detection (BIRD) satellite over fires in Benin (West Africa) in December 2002 and over Portugal in August 2003, as already used in [15].

The spectral bands of the BIRD MIR and TIR channels are similar to those of MTG-I/FCI (see Table 7). BIRD had a spatial resolution of 370 m with a sampling step of 185 m (due to the used staggered line array detectors and double sampling, used in the HSRS of BIRD (and also in the IR sensors of TET-1 and BIROS), the ground sampling step is ½ of the linear pixel size, which is, for instance, explained in detail in [6]) and a high saturation level of ~600 K, allowing an adequate simulation of MTG-I/FCI images. For the simulation, a 1 km sampling mode and a 2 km sampling mode of the MTG-I/FCI MIR/TIR fire application channels were assumed.

**Figure 8:**
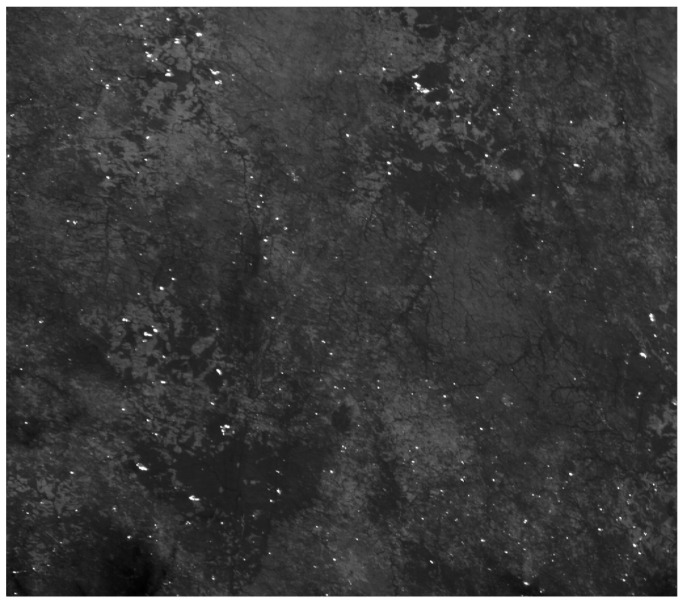
Fragment of the BIRD MIR image from the Benin fire scene from 1 December 2002.

**Figure 9:**
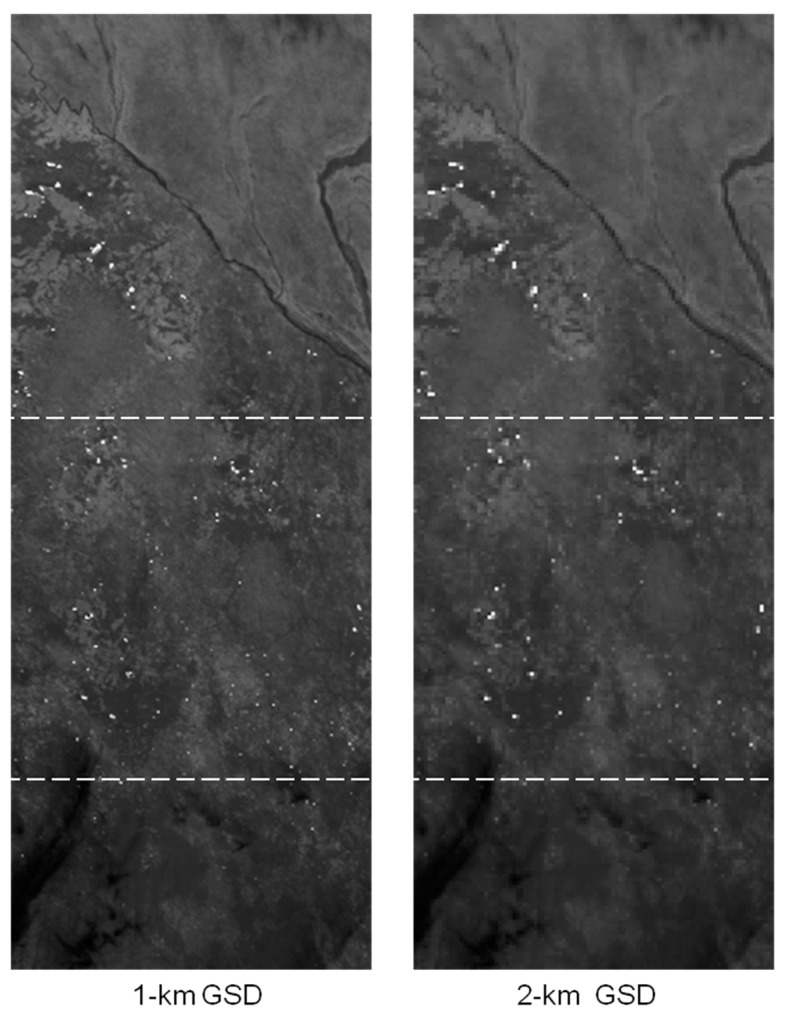
Simulated MTG-I/FCI MIR image fragments with 1 km and 2 km GSDs. The original BIRD image fragment shown in Figure 8 corresponds to the center squares of these two images.

Numerous fires in Benin were imaged by BIRD on 1 December 2002. The scene, shown partly in Figure 8, is dominated by small fires. The scene has 1024 × 2700 BIRD pixels with its center at 9.3° N, 1.8° E. The expected resolution of the MTG-I/FCI at this latitude is approximately 1.0 × 1.0 km^2^ in the 1 km mode and 2.0 × 2.0 km^2^ in the 2 km mode (see Table 8). The MTG-1/FCI signals were simulated by aggregating 5 × 5 and 10 × 10 BIRD pixels. The simulated MTG-I/FCI MIR images are shown in Figure 9.

The pixel sizes and the pixel area for the MTG-I/FCI MIR/TIR fire application channels in the 1 km mode and in the 2 km mode are presented in Table 8 as a function of the latitude, considering the pixels in the nadir part of the east–west scans (the pixel sizes and areas at the scan edges of MTG-I/FCI will be larger).

All hot pixel signals in the simulated MTG-I/FCI Benin image are far from the FCI “maximum signal” temperatures for the fire application channels—presented in Table 7—both in the 1 and 2 km sampling modes, i.e., the respective maximum pixel temperatures simulated for the Benin scene are 401 K or 361 K in the MIR channel and 318 K or 315 K in the TIR channel.

The fire detection potential of a sensor can be characterized by the minimum FRP of the hot clusters. While BIRD data allow detecting fires with an FRP as low as 0.3 MW, the minimum FRP of the detected fires for MTG-I/FCI in the simulated scenes was 6.6 MW in the 1 km sampling mode and 48.6 MW in the 2 km sampling mode, using the FRP equation, Equation (1), of Section 2.2 of this paper.

As a result, significantly fewer hot clusters could be detected in the simulated MTG-I/FCI images (122 in the 1 km mode and 48 in the 2 km mode) in comparison to 516 hot clusters which were detected by BIRD (see Table 8 below). Nevertheless, the decrease in the total FRP summarized for all detected fires in the whole scene is not so significant: 11.0 and 9.6 GW for the 1 or 2 km MTG-I/FCI sampling modes compared to 11.7 GW for BIRD. This result can be explained by the following two effects:

(1) The overwhelming part of the total FRP, summarized for the whole scene, is contributed by large fires that are also detectable in an MTG-I/FCI fire application MIR band pixel;
(2) Due to the BIRD MIR band pixel aggregation conducted for the MTG-I/FCI MIR band simulation, a part of the small fires in the BIRD MIR band data is not omitted during this pixel aggregation but rather “collected” in larger hot clusters providing enough signal in the simulated MTG-I/FCI MIR band scene to retrieve an FRP estimate. This way, even such “small fire ensembles” in the initial BIRD scene can still contribute to the total FRP derived and summarized for the whole scene during the MTG-I/FCI simulation.

The retrieval errors are defined—as described in [3]—in relation to the TIR background clutter, which is the principal effect introducing error to the bi-spectral retrieval for hot clusters with a fire proportion less than 0.001, i.e., in pixels occupied by fire at less than 0.1%. This is the case for BIRD data and for the simulated MTG-I/FCI observations, too.

The simulated MTG-I/FCI data (i) of the Benin scene and (ii) of a fire scene in the Castelo Branco region of Portugal allowed the retrieval of the effective fire temperature and area for a much smaller number of hot clusters than in the respective original BIRD scenes. To secure a quantitative comparison of the simulation results, the following comparable accuracy conditions were applied for the estimation of the three attributes fire temperature, fire area and FRP:

- Accuracy of 100 K for the fire temperatures;
- An accuracy “factor of 2” for the fire areas (an accuracy “factor of 2” for the fire areas means that the area is estimated with an uncertainty, i.e., the exact fire area may be lower by a factor of 2 or higher by a factor of 2 than the estimated fire area);
- Thirty percent FRP accuracy (a “30% FRP estimation accuracy condition” means that the FRP is estimated with an uncertainty, i.e., the exact value of the FRP may be 30% lower or 30% higher than the estimated FRP value).

These conditions are actually not the requirements set up by the user (who would probably prefer much better accuracies), but rather the retrieval accuracies that can reasonably be expected from the satellite data-based bi-spectral retrievals [3].

The total number of hot clusters with the three fire attributes—as presented in the three right columns of Table 9 for the BIRD data, the simulated MTG-I/FCI data and the MODIS data—decrease for the simulated MTG-I/FCI data and MODIS data. Meanwhile, the BIRD data meet the retrieval accuracy conditions for 16–19% of the detected hot clusters; the retrieval accuracy conditions decrease to 10% in the 1 km sampling mode simulation for MTG-I/FCI; and all detected hot clusters do not meet the “factor-2” fire area accuracy conditions in the 2 km sampling mode simulation for MTG-I/FCI.

Nevertheless, the simulation shows that MTG/FCI will meet the applied “30% FRP estimation accuracy condition” for two out of every three hot clusters in both sampling modes.

**Figure 10:**
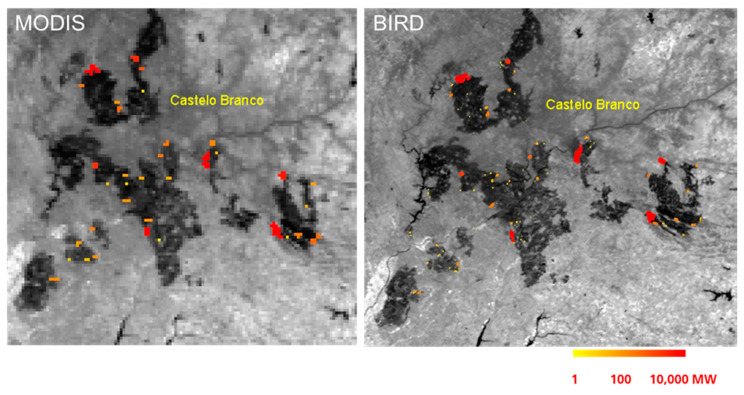
Two MIR band image fragments of a fire scene in Portugal obtained on 4 August 2003 by MODIS (**left**) and BIRD (**right**) with ground sampling distances of 1 km and 180 m, respectively, and with color-coded FRPs of the fire clusters.

A second BIRD data example selected for this MTG-I/FCI simulation was a large fire scenario in the Castelo Branco region of Portugal observed on 4 August 2003. Figure 10 shows two MIR band image fragments of a fire scene in Portugal obtained on 4 August 2003 by MODIS “Terra” (left) and BIRD (right) with ground sampling distances of 1 km and 180 m, respectively, and with color-coded FRPs of the fire clusters. The MODIS image was obtained 34 min earlier than the BIRD image that was used for the MTG-I/FCI image simulation. Though the general distribution of burning in both images of Figure 10 is similar, one can also notice differences in the location and intensity of some hot spots due to the time interval between the images.

The proportion of big fires in this scene was much higher than in the Benin case. The BIRD image fragment has 600 × 800 pixels, and its center is located at 40° N, 7.6° W. The expected spatial resolutions of MTG-I/FCI at this latitude are 1.06 × 1.58 km^2^ in the 1 km sampling mode or 2.12 × 3.16 km^2^ in the 2 km sampling mode, and 5 × 8 or 10 × 16 BIRD pixels are aggregated for the 1 km pixels or 2 km pixels of MTG-I/FCI, respectively.

The MTG-I/FCI fire application channel simulation results are summarized in Table 9.

**Figure 11:**
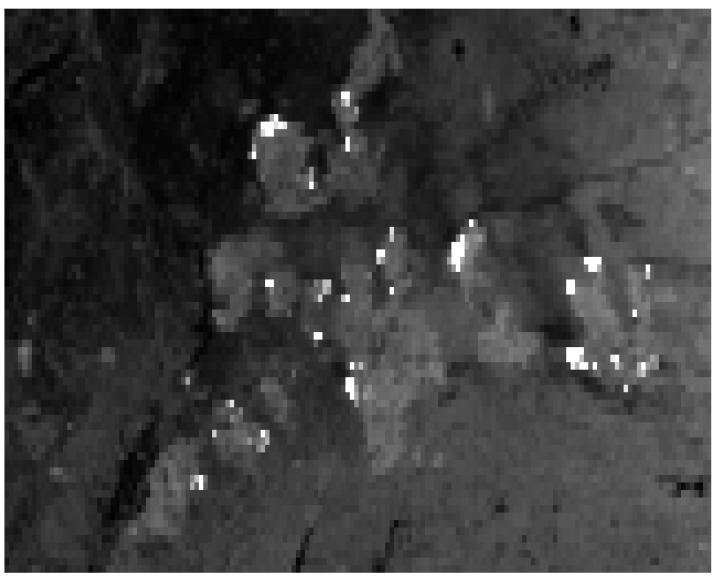
Simulated MTG-I/FCI 1 km sampling mode MIR image using the BIRD image obtained over the Castelo Branco region in Portugal on 4 August 2003 at 12:04 h GMT.

The small difference between 35 hot clusters detected in the MODIS image fragment (Figure 10, left image) and 30 hot clusters detected in the simulated MTG-I/FCI 1 km resolution image fragment (Figure 11) can be explained by the slightly higher spatial resolution of MODIS compared to the simulated MTG-I/FCI resolution in the 1 km mode, which results in a pixel size of 1.06 × 1.58 km over Portugal. However, MODIS data permit calculating a total, i.e., aggregated over the whole scene, FRP of only 12.0 GW for this scene. This is low compared with the total FRP of 15.9 GW in the simulated MTG-I/FCI image with the 1 km sampling mode.

A total of 2 out of 97 hot pixels in the simulated MTG-I/FCI image in the 1 km sampling mode slightly exceed the 450 K saturation level of its MIR channel, while in the 2 km sampling mode, all MIR pixel temperatures are below 422 K. The hot pixels in the MTG-I/FCI TIR channel remain again far from the saturation level of 400 K for both the 1 km and 2 km sampling modes.

The analysis of the bi-spectral retrievals for fire attributes leads to similar conclusions to the Benin fire scenario for the Portugal fire scenario (see Table 9):

- In spite of the fact that MTG-I/FCI detects much fewer active fires than BIRD, due to the omission of weaker or smaller fires, it registers nearly the same total FRP.
- The total FRP value differences from BIRD are still smaller for the Portugal fire scenario (15.9 and 15.2 GW for the 1 and 2 km MTG-1/FCI sampling modes compared with 15.9 GW for BIRD), since the proportion of large fires in Portugal was higher than in Benin.
- In the 1 km sampling mode, the simulated MTG-I/FCI MIR and TIR channel data allow the retrieval of the following, both for a much smaller number of hot clusters, compared with the BIRD data:
  ○ The effective fire temperature applying the accuracy condition of 100 K;
  ○ The effective fire area applying the “factor of 2” accuracy condition.
- However, in the 2 km sampling mode, the simulated MTG-I/FCI MIR and TIR channel data do not meet these applied accuracy conditions for all the detected hot clusters.

Nevertheless, for the Portugal and Benin fire scenarios, the “30% FRP accuracy” condition is achieved for two out of every three hot clusters in both MTG-I/FCI sampling modes, which corresponds to the 63–67% in the outer right column of Table 9.

The performed simulation allows the following assessment of the fire detection and analysis capability of the MTG-I/FCI fire application channels for typical African and European wildfire scenarios (such as the Benin and Portugal scenes):

- The specified dynamic range of the MTG-I/FCI MIR channel—with a 450 K saturation temperature (see “Maximum Signal”, in Table 7 for fire application channels)—would allow:
  ○ Unsaturated imaging of all the fires in the 2 km mode;
  ○ Nearly unsaturated imaging in the 1 km mode (only 2 out of 97 hot pixels in the Portugal fire scene had an MIR channel pixel temperature of 460–470 K, i.e., higher than the expected saturation level at 450 K).
- The MTG-I/FCI TIR channel—with a 400 K saturation temperature—will be far from saturation for all the fires.
- MTG-I/FCI fire application MIR and TIR channel data will allow for detection of nearly all fires in the 1 km sampling mode which are also detected by MODIS, but it will miss most of the small fires (usually detected by BIRD, FireBIRD or VIIRS) unless they are located close together—together occupying one MTG-I/FCI pixel. Nevertheless, in fire scenes with intensive burning, the MTG-I/FCI data will underestimate the total accumulated FRP by 0–4% only in the 1 km sampling mode and by 6–18% in the 2 km sampling mode, depending on the fire size distribution. (The FRP underestimation may be more significant in the scenes dominated by small fires.)
- MTG-I/FCI fire application channel data will probably fulfill the “30% FRP accuracy” condition for two out of every three detected active fires in both MTG-I/FCI sampling modes for typical wildfire scenarios.
- MTG-I/FCI fire application channel data will permit an estimation of:
  ○ The effective fire temperature with an accuracy of 100 K;
  ○ The effective fire area (i) for only 7–17% of the detected active fires in the 1 km sampling mode (with an area accuracy “factor of 2”) and (ii) only in exceptional cases in the 2 km sampling mode.

## 5. Capability of FireBIRD Class Data for Validation of the Fire Radiative Power Obtained from MTG-I/FCI—Class IR Data

In Table 9 of the previous chapter, minimum FRP values of 48 or 96 MW for the detected fires in the simulated MTG-I/FCI images in the 2 km sampling mode for the Benin or Portugal scene, respectively, are presented. The simulated MTG-I/FCI 2 km mode corresponds to the GSD of the Himawari-8 AHI at its sub-satellite point (SSP).

These values confirm the statement of [13] that “…AHI underestimates FRP by around 50% compared to the simultaneous MODIS view, because it cannot easily detect the (rather common) fires whose FRP lies below ~40 MW”.

MTG-I/FCI will provide unsaturated MIR and TIR fire application channel data of wildfires in Africa and Southern Europe within a time interval of 10 min with two selectable GSDs at an SSP of 2 km or 1 km.

The ground resolution of 1 km for the FCI at the SSP of MTG-I is comparable to the resolution of fire application channel data of MODIS and SLSTR, and it will be a little bit coarser than the VIIRS M-band fire product with a 750 m GSD.

Therefore, one can assume that an improved quality of fire observation and quantitative evaluation of fires in their diurnal cycle can be achieved if:

- MIR and TIR band data acquired by MTG-I/FCI within time intervals of 10 min with ground resolutions of ~1 km can be used to derive the Fire Radiative Power (FRP) of medium and larger fire clusters—every 10 min and covering their whole diurnal cycle;
- MIR and TIR band data acquired several times per day with ground resolutions of ~200 m, for instance, by BIROS, or by thinkable prospective satellites of the FireBIRD class, are used to derive the FRP of these medium and larger fire clusters with higher accuracy;
- These FRP figures with a higher estimation accuracy are used to augment or validate the FRP derived from geostationary satellite 1 km resolution data—as it is illustrated in Figure 5 (copied from [13]) for data from MODIS and Himawari-8;
- The FRP values of these selected and validated fire clusters can serve as local reference points for the FRP figures obtained by MTG-I/FCI within 10 min intervals, which should then be integrated into Fire Radiative Energy (FRE) figures—for each fire cluster over its whole time of burning;
- These FRE figures are used to estimate the amount of carbon, i.e., CO_2_, CO, particulate matter (PM) and black carbon (BC), released by each fire cluster during its live time.

The possibility to calculate the FRE over the fire diurnal cycles may significantly improve, for instance, the estimation accuracy of Dry Matter Burned (DMB), an important input parameter for the Global Fire Assimilation System (GFAS) [11,13].

## 6. Conclusions and Outlook

The IR sensors of BIRD, TET-1 and BIROS provided image data in the mid-IR (MIR, at 3.8 µm) and thermal IR (TIR, at 9.2 µm) bands with ground sampling distances (GSD) of ~180 m in both and noise equivalent difference temperatures (NEDT) of 0.4 K (at 300 K) in the MIR band and 0.2 K (at 300 K) in the TIR band, and they comprise a very high radiometric dynamic in both bands, necessary to avoid signal saturation from strong high-temperature events (HTEs), such as large wildfires or major volcanic eruptions.

The IR sensors of BIRD and FireBIRD delivered more detailed, locally based information on fire occurrence than the operational IR sensors currently used for fire applications, and they allowed fire dynamic measurements that were previously not possible.

The BIRD and FireBIRD missions offered an opportunity to monitor the true fire situation from space and to estimate attributes or characteristics of small and large fires, such as the Fire Radiative Power from 0.3 MW upward, the effective fire temperature, the effective fire area, the fire front length and the radiative fire front intensity, i.e., the ratio of the FRP to the front length of a fire cluster.

The provision of active fire information products requires data records of appropriate bi- or multi-spectral IR imagery, for example, through the following classes of instruments and satellites:

- A set of geostationary meteorological satellites, providing frequent but lower-spatial resolution MIR/TIR fire application-relevant data with a ground resolution of 2 km or better at their sub-satellite points (SSPs);
- Polar orbiting satellites with instruments of the MODIS, SLSTR and/or VIIRS class, operated in a “24/7” mode;
- FireBIRD-type IR instruments operated in a small satellite constellation, providing:
  ○ Higher-spatial resolution data acquisition without signal saturation from large fires;
  ○ An augmentation and validation possibility of the more frequent but lower-spatial resolution active fire datasets, provided by the systems mentioned above, for detecting smaller and weaker fires, including the derivation of their FRP values;
  ○ Higher-spatial resolution MIR and TIR band data of fires (a) at different local times and (b) at afternoon local time when fires are at a maximum (see Figure 5).

Except for the ECOsystem Spaceborne Thermal Radiometer Experiment on Space Station (ECOSTRESS) (see https://ecostress.jpl.nasa.gov (accessed on 1 December 2021) with six spectral bands in the TIR band (with pixel saturation temperatures at ~380 K) and the proposed Dynamic Infrared Earth Observation on ISS Orbit (DIEGO) in Germany (see [16]), proposed with an MIR band and several TIR bands, there are no existing or planned multi-spectral mid and thermal IR missions delivering higher-spatial resolution (<100 m) data at varying times of the day, including repeated acquisitions at afternoon local times.

It should be stressed that this type and the varying and afternoon local times of measurements are very essential for many climate change-related applications, such as urban heat islands (UHI), epidemiology, evapotranspiration and water stress of plants and water management.

For these climate change-related applications and for higher-spatial resolution fire monitoring, there are very similar requirements with regard to the radiometric and spatial resolution of IR imaging data records conducted at different local times and at the afternoon local time (including short revisit times).

The “Copernicus Space Component (CSC) Long Term Scenario”, issued by the ESA Earth Observation Program Board in May 2019, states in its chapter “Optical Imaging Family”, and its sub-chapter “Evolution of Capabilities” on page 12 for higher-resolution thermal Earth observation:

*“For TIR imaging, the current assumption is to deploy a constellation of TIR satellites flying in orbits coordinated with those of Sentinel-2 NG mission and chosen in such a way that parameters for agriculture management are adequately observed. The user requirements in respect of land surface temperature lead to a wide swath to enable fast revisit, whereas accuracy and spatial resolution need to suit European agriculture fields and need to be consistent with Sentinel-2. A mid size satellite carrying a thermal infrared wide-swath sensor together with a low performance camera in the visible and near infrared for corrections and interpretation can be assumed. However, the revisit required can be achieved only with multiple satellites and could be further enhanced through small satellites complementing the observations of medium-size satellites.”*

Actually, the creation of an infrared monitoring constellation (IR-MC) should be considered, consisting of several dedicated small satellites of the FireBIRD class, operated in low Earth orbit (LEO) and equipped with compact sensors which deliver (preferably twice a day at different local times including the afternoon) higher-spatial resolution mid and thermal IR data in near-real time for UHI, epidemiology, evapotranspiration of plants, water management and wildfires, complementing the observations of medium-sized operational polar orbiting satellites.

The IR-MC data products, including maps with fire attributes, such as the FRP, should be provided in photogrammetric mapping standards to permit the use of these products together with other information in a geographic information system (GIS).

## Figures and Tables

**Table 1:** Geometric features and further sensor parameters of the operational IR sensors MODIS, VIIRS and SLSTR in comparison with HSRS, flown on BIRD, TET-1 and BIROS.

Feature/Parameter	MODIS	VIIRS	SLSTR	HSRS
Launched in the year with *Satellite*	1999 “*Terra*”, 2002 “*Aqua*”, i.e., two nearly identical instruments	2011 “*Suomi NPP*”, 2017 “*NOAA-20*”	2015 “*Sentinel-3A*”, 2018 “*Sentinel-3B*”	2001 “*BIRD*” 2012 “*TET-1*” 2016 “*BIROS*”
Orbit altitude	705 km	829 km	815 km	500–560 km
Swath width	2330 km	3060 km	1407 km	162–178 km
Ground sampling distance (at the nadir)	1000 m	375 m	1000 m	170–185 m
MIR and TIR pixel saturation temperature ^(1)^	MIR: 450 K, TIR: 400 K	MIR: 367 K, TIR: 300 K	MIR: 500 K, TIR: 400 K	MIR: 630 K TIR: 600 K
Minimum detectable area of a 1000 K THE ^(2)^	~150 m^2^	~20–30 m^2^	~150 m^2^	15–20 m^2^
Revisit time	12 h (achived with two instruments)	12 h	24 h	12 h–3 d ^(3)^

^(1)^ In the IR bands used for observation of high-temperature events (HTEs), ^(2)^ at 300 K background temperature, see also Figure 15a in [3] for more details. ^(^^3)^ The revisit time of BIRD, TET-1 and BIROS is/was variable due to the possibility to move the line of sight (LoS) by +/− 30° from the nadir. This “roll-movement” of the LoS is a tool to enhance the field of regard (FoR) of the satellite sensor. This also allows observing an area of interest (AoI) three times within 3 days.

**Table 2:** The LTAN and LTDN of the operational satellites carrying the IR sensors MODIS, VIIRS and SLSTR, as well as the LTAN and LTDN of BIRD, TET-1 and BIROS.

Satellite (IR Sensor)	Launch Date	LTDN	LTAN	Comment	“Grouping” N°
Terra (MODIS)	1999	10:30 h			1
BIRD	2001	10:30 h (2001) 11:30 h (2003)		LTDN changed in 2 years by ~1 h	
Aqua (MODIS)	2002		13:30 h		2
TET-1	2012		11:30 h (2012) 12:30 h (2015)	LTAN changed in 3 years by ~1 h	
Suomi NPP (VIIRS)	2011	10:30 h			3
Sentinel-3A (SLSTR)	2016	10:00 h			
BIROS	2016	10:30 h (2016) 10:15 h (2018)		LTDN corrected by propulsion system	
NOAA-20 (VIIRS)	2017		13:30 h		2
Sentinel-3B (SLSTR)	2018	10:00 h		Phased by 140° to Sentinel-3A	3

**Table 3:** Overview of some scientific papers and documents, structured by “grouping” numbers.

“Grouping” N°	Reference	Title (and Main Content) of the References
1	Wooster et al., 2003 [8]	Fire radiative energy for quantitative study of biomass burning: Derivation from the BIRD experimental satellite and comparison to MODIS fire products
	Zhukov et al., 2006 [3]	Spaceborne Detection and Characterisation of Fires during the Bi-spectral Infrared Detection (BIRD) Experimental Small Satellite Mission (2001–2004)
	Oertel et al., 2005 [9]	BIRD Fire Recognition and Comparison with MODIS/Terra
2	Atwood et al., 2016 [10]	Detection and Characterization of Low Temperature Peat Fires during the 2015 Fire Catastrophe in Indonesia Using a New High Sensitivity Fire Monitoring Satellite Sensor (FireBIRD)
3	Nolde et al., 2021 [11]	The DLR FireBIRD Small Satellite Mission: Evaluation of Infrared Data for Wildfire Assessment (using FireBIRD, MODIS and VIIRS data, registered over six fire scenes)
	Soszynska, 2021 [12]	Parametrisation of Gas Flares Using FireBIRD Infrared Satellite Imagery, Thesis: “Analysis of gas flaring observed from space using remote sensing satellite imagery from FireBIRD, VIIRS on Suomi NPP, SLSTR on Sentinel-3A and further satellite sensors”
	Halle et al., 2020 [5]	Infrared-Image Processing for the DLR FireBIRD Mission, chapter on: “Image observation of the devastating “Paradise fire” in November 2016 with TET-1, BIROS and VIIRS”

**Table 4:** Attributes of numbered forest fire fronts in the BIRD image fragment in Figure 1 (right) of the Lake Baikal area in Siberia, Russia, obtained on 16 July 2003.

Fire Cluster (Number in Figure 1, BIRD)	Fire Radiative Power (FRP) (MW)	Fire Front Length (km)	Fire Front Strength (kW/m)	Fire Front Effective Depth (m)
1	1829	8.2	223	7.7
2	150	5.8	26	1.9
3	409	6.5	63	3.2
4	111	4.8	23	1.1
5	126	3.4	37	1.3
6	568	5.0	114	3.8
7	136	6.3	22	1.2

**Table 7:** Characteristics of the MTG-I/FCI fire application channels [14].

Channels	Centrum Wavelength (µm)	Band-Width (µm)	GSD at SSP (km)	Minimum Signal (K)	Maximum Signal (K)	NEDT (K)
FD-IR 3.8 ^#1,#2^	3.8	0.40	2 or 1 ^#1^	200 350 ^#2^	350 450 ^#2^	0.1–0.2 ^#1^ 1 ^#2^
FD-IR 8.7 ^#1,#2^	8.7	0.30	2 or 1 ^#1^	165 330 ^#2^	330 400 ^#2^	0.1 0.5 ^#2^

^#1^ Figures for the FCI channels to be delivered in both the High Resolution and Fast Imagery (HRFI) mode and Full Disk High Spectral Resolution Imagery (FDHSI) mode (together with the NEDT figures applicable for the HRFI mode). ^#2^ These figures represent the fire application channels with extended dynamic ranges, the reference temperature and relaxed noise requirements applicable for this application. The NEDT figures are applicable to the complete extended radiometric dynamic range.

**Table 8:** MTG-I/FCI ground pixel sizes (west–east × south–north) and area (km^2^) as a function of the latitude.

	Latitude					
	0°	10°	20°	30°	40°	50°
1 km mode:						
pixel size	1.00 *×* 1.00	1.00 *×* 1.03	1.02 *×* 1.12	1.04 *×* 1.29	1.06 *×* 1.58	1.09 *×* 2.12
pixel area	1	1.03	1.13	1.33	1.67	2.31
2 km mode:						
pixel size	2.00 *×* 2.00	2.01 *×* 2.05	2.03 *×* 2.23	2.07 *×* 2.57	2.12 *×* 3.16	2.18 *×* 4.23
pixel area	4	4.13	4.53	5.14	6.69	9.23

**Table 9:** MTG-I/FCI fire application channel simulation results for the Benin and Portugal fire scenes, where the three right “error columns” show the numbers or the percentage (%) of the total number of detected fires with: (i) a fire temperature estimation error less than 100 K, (ii) fire area estimates with an accuracy “factor of 2”, i.e., the exact areas may be by a factor of 2 lower or higher than the estimated area values, and (iii) FRP values with estimation errors less than 30%.

Dataset (^r^—Real Image; ^s^—Simulated Image)	Number of Detected Hot Clusters	Minimum Detected FRP *(MW)*	Total FRP *(GW)*	Number of Hot Clusters with:		
				Fire Temperature Error Less than 100 K	Fire Area Error Factor Less than 2	FRP Error Less than 30%
Benin:						
BIRD (0.37 km) ^r^	516	0.3	11.7	82 (16%)	98 (19%)	384 (74%)
MTG 1 km mode ^s^	122	6.8	11	12 (10%)	12 (10%)	80 (66%)
MTG 2 km mode ^s^	48	48.6	9.6	4 (8%)	0	32 (67%)
Portugal:						
BIRD (0.37 km) ^r^	93	0.54	15.9	19 (20%)	27 (29%)	64 (69%)
MTG 1 km mode ^s^	30	27.6	15.9	2 (7%)	5 (17%)	19 (63%)
MODIS (1 km) ^r^	35	16.7	12	0	4 (11%)	22 (63%)
MTG 2 km mode ^s^	19	96.6	15.2	0	0	12 (63%)
SEVIRI (3 km mode) ^s^	9	372	14.3	0	0	6 (67%)

## Data Availability

FireBIRD data are free of charge and publicly available via DLR’s open data archive EOWEB (https://eoweb.dlr.de/egp/, assessed on 9 December 2021).
